# Infant Mortality Screening and Prevention Initiatives in Nepal

**DOI:** 10.7759/cureus.52366

**Published:** 2024-01-16

**Authors:** Mahi Basra, Saajan Patel, Diksha Nepal, Cyril Blavo

**Affiliations:** 1 Medicine, Nova Southeastern University Dr. Kiran C. Patel College of Osteopathic Medicine, Clearwater, USA; 2 Nephrology, National Academy of Medical Sciences, Kathmandu, NPL; 3 Pediatrics, Nova Southeastern University Dr. Kiran C. Patel College of Osteopathic Medicine, Clearwater, USA

**Keywords:** national neonatal health strategy, community based integrated management, nepal screening programs, nepal infant mortality, child mortality, paediatric screening, neonatal mortality, screening, pediatric screening, nepal

## Abstract

Infant mortality is one of the leading public health crises in Nepal. While Nepal has made significant advances in mitigating under-five mortality, much work is still needed to be done regarding the healthcare of infants. The Nepalese government has identified this as a problem and has introduced a series of interventions to improve the health outcomes of infants. The aim of this review is to identify the goals, interventions, and effectiveness of major infant mortality prevention programs around the country. A comprehensive literature search was performed using PubMed and Google Scholar. The literature search revealed six programs that Nepal has utilized to combat infant mortality.

The Community Based Management of Childhood Illness (CB-IMCI) program utilizes specially trained community workers to help identify and treat children with common childhood illnesses. The National Neonatal Health Strategy (NNHS) links families to the community and then to the broader healthcare system, with success found in its referral system. The Safe Delivery Incentives Program (SDIP) has found success with monetizing safe delivery practices, and shown an increase in safe deliveries with skilled healthcare workers present. Free Newborn Care (FNC) services were aimed at treating sick newborns for free, but ongoing concerns for program sustainability have led to further revision. The Every Newborn Action Plan (ENAP) is another plan aimed at preventing newborn deaths through improving health system administration and finances, but with limited efficacy data, it is hard to determine its success due to the lack of objective benchmark markers and data collected. Finally, the Birth Preparedness Package (BPP) is a highly efficacious program that encourages communities to plan for pregnancies by planning for delay barriers. Nepal has made significant strides in reducing infant mortality; however, much work still needs to be done. From 1990 to 2020, Nepal has reduced the under-five mortality rate from 138.8 deaths per 1,000 live births to 28.2 deaths per 1,000 live births.

## Introduction and background

Nepal has been described as one of the least developed nations in the world [1]. Situated in the Himalayas, between India and China, Nepal is divided into four major regions: Tarai, Churia, Mahabharat, and the Great Himalaya range [1]. The majority of the Nepalese population is comprised of migrants from Tibet or of Indo-Aryan heritage [1]. Nepal’s economy is heavily dependent on imports due to its landlocked geography and inadequate transportation [1]. The government has several development programs geared toward addressing poverty, hunger, the need for education, and the requirement for clean water, often funded by foreign aid [2].

Neonatal mortality is defined as infant death within the first 28 days of life [3]. Almost half of deaths in children under five years occurred within the newborn period in 2020 [3]. Specifically, 2.4 million newborns died worldwide in 2020, attributed to a lack of quality healthcare, during the first two to three days of life [3]. This amounts to approximately 6,700 deaths every day [3]. Over 75% of neonatal deaths occur within the first seven days of life worldwide [3]. Globally, 28% of neonatal deaths have been attributed to preterm birth: 26% to severe infections, 23% to asphyxia, and 7% to neonatal tetanus [4]. The United Nations International Children's Emergency Fund (UNICEF) noted the preventable nature of these tragic deaths and launched A Promise Renewed in 2012 and Every Newborn Action Plan in 2014 [5]. Sustainable Development Goals (SDGs) were set for these initiatives, aimed at preventing deaths of newborn children and those younger than five years by 2030 [5]. Nepal, a member of the United Nations, aims to implement these SDGs by upholding the leave-no-one-behind principle [2]. Nepal has made significant progress in mitigating under-five mortality represented by the Millennium Development Goal 4; however, there are substantial advances to be made regarding neonatal and child care [4].

Infant mortality is among the leading public health concerns in Nepal [6]. Nearly 50,000 infants under the age of one die annually, and there are over 30,000 neonatal deaths per day [6]. The Ministry of Health and Population (MOHP) of Nepal and the WHO have recognized infant mortality as a leading cause of preventable deaths and have employed a series of interventions to improve the health of infants. The purpose of this review is to explore various health interventions in Nepal aimed at addressing preventable infant mortality. A comprehensive literature search was performed to identify the goals, interventions, and effectiveness of programs around the country. The programs evaluated include Community Based Integrated Management of Childhood Illness, National Neonatal Health Strategy, Safe Delivery Incentives Program, Free Newborn Care Services, Every Newborn Action Plan, and the Birth Preparedness Package. These programs represent the major infant mortality prevention initiatives in Nepal. This review hypothesizes that each of these programs provides a vital step in reducing infant mortality in Nepal.

## Review

Methods

A comprehensive literature search was performed using PubMed and Google Scholar for articles from the years 2004-2023. Key search terms included “newborn health screening”, “community-based integrated management of childhood illnesses in Nepal”, “national neonatal health strategy”, “safe delivery incentives program”, “free newborn care services”, “every newborn action plan”, “birth preparedness package”, “neonatal health screening in Nepal”, “infant health screening in Nepal”, “neonatal mortality”, and “infant mortality”. Abstracts were screened and were excluded if they were not written in English, were animal studies, were poster presentations, and were not programs implemented in Nepal. The remaining articles' full text was read and reviewed, and duplicates were removed.

Community-Based Integrated Management of Childhood Illness (CB-IMCI)

The CB-IMCI is a program in Nepal that has followed earlier community intervention programs such as the Control of Diarrheal Disease (CDD) program and the Acute Respiratory Infection (ARI) program [7]. The CB-IMCI is implemented through the initiative of the WHO and UNICEF to improve child health globally, known as the Integrated Management of Childhood Illness (IMCI) [8]. IMCI has three main goals: improving the capability of child healthcare practitioners, improving the organization and efficiency of healthcare services, and increasing awareness of best care practices [8]. Nepal has adopted tenets of IMCI, initiated in 1997, through the use of female community health volunteers (FCHVs), community health workers (CHWs), and village health workers (VHWs) [8]. Focusing on community-wide pneumonia treatment, these workers undergo five to seven days of intensive training [7]. This training prepares workers to identify children with pneumonia by utilizing a unique respiratory rate timing system and provide treatment based on the WHO algorithm for IMCI [7]. A mother’s group orientation is provided in each community. Caregivers bring their ill children to the CHWs, who assess the patient for warning signs and symptoms and may treat them based on the WHO algorithm. CHWs are trained to treat pneumonia in children between two months and five years of age with clotrimazole pediatric tablets or refer severe cases to healthcare centers. CHWs are also trained to treat diarrhea in children with adequate hydration and zinc tablets [7]. Nepal was one of the first countries to introduce zinc administration in public districts for diarrhea treatment through financial support from the national government and the United States Agency for International Development (USAID).

Efficacy measurements have shown that the community-based pneumonia treatment protocol within CB-IMCI facilitated treatment for 60% of pneumonia cases in the districts of Nepal [8]. When compared to the 30% treated in non-CB-IMCI districts, a clear improvement in health outcomes can be seen [8]. A program management package was added to encourage CHWs to evaluate logistics, provide supervisory visits, and conduct review meetings to discuss challenges and achievements [7]. A monitoring system was also established to compile service data to report to the child health division of the Nepal MOHP [7]. This system was not a part of the original WHO IMCI approach, but was added by the MOHP to analyze effectiveness. The impact of the CB-IMCI was evaluated in 2006-2007 prior to the expansion of the program to additional districts. Reports displayed a decrease in diarrheal dehydration and ARI case incidence in districts with interventions [9]. This demonstrates that Nepal has successfully trained several FCHVs to recognize health warning signs and is working toward a decrease in child mortality in alignment with the SDGs [9].

National Neonatal Health Strategy (NNHS)

Recognizing the need for a reduction in neonatal mortality, the Nepal MOHP developed the NNHS in 2004 [7]. The Nepal MOHP acknowledged that every vulnerable newborn had the greatest right to be taken care of and subsequently implemented a strategy to improve the health and survival rates of the newborns [10]. This strategy was developed on the basis of a detailed analysis of neonatal health in 2002 and a series of papers that were published by the Neonatal Health Working Group [11]. By focusing on evidence-based practices, evaluation of cost-effectiveness, and innovative approaches, the strategy aims to improve the health of the community at a local and national level. The NNHS aimed to have an immediate impact by establishing a chain of care, linking families to the community, and then to the health system. The long-term goals of NNHS, outlined in Table 1, include strengthening health promotion, expanding cost-effective newborn initiatives, improving local programs, and prioritizing policy to implement behavior change and enhance health service delivery [11].

**Table 1:** Goals of the NNHS program NNHS: National Neonatal Health Strategy Adapted from [11]

Changes to be implemented in NNHS	Goals of change
Policy changes	Establish neonatal group within the Safe Motherhood Subcommittee; facilitate collaboration between communities; facilitate standardization and institutionalization of neonatal care as a formal specialty and provide training for secondary and tertiary health care workers; assemble the National Breastfeeding Promotion and Protection Committee; establish universal registration of births and deaths
Behavioral changes	Develop a National Neonatal Birth Control Package to promote newborn care that integrates national policies and strategies; disseminate mass media education targeted toward women, husbands, and mothers-in-law to encourage proper care of pregnant women and newborns (emphasis is placed on antenatal visits, rest, nutrition, skilled birth attendance); educate on newborn care practices such as clean umbilical cord cutting, drying, wrapping, and delaying bathing; conduct further testing of the birth preparedness package; bolster school and community-wide reproductive health education; introduce kangaroo mother care; advocate for mothers and children
Strengthening health service delivery	Increase skilled birth attendant attendance at delivery; develop effective training for the NNHS workers; train non-formal caregivers such as family and friends on safe newborn packages; Emphasize the importance of at least three postnatal visits within 24-48 hours, within six days and again at six weeks; promote kangaroo maternal care; strengthen the National Tetanus Elimination Program for both mothers and newborns; establish an effective health referral program to allow for timely transfer of sick newborns to a health facility

The plan of the NNHS is to establish neonatal care at four levels: (1) home and community level, (2) primary healthcare, (3) district hospitals, and (4) central and regional hospitals [10].

Home-level care involves family and community awareness of appropriate newborn care, danger signs of newborn mortality, and reduction of harmful practices through promotional messages and tools. These strategies are designed to be implemented by FCHVs and CHWs [10]. Notable goals include at least three antenatal checkups, skilled attendant during delivery, awareness regarding clean cord delivery, assurance of a warm delivery place, recognition of asphyxia, initiation of immediate breastfeeding, identification of simple infections and jaundice, recognition of premature newborns, and access to appropriate care [10]. CHWs are trained to understand the causes and prevention of hypothermia, resuscitation of asphyxiated babies (through mouth-to-mask ventilation), and home management of simple infections with antibiotics such as clotrimazole or gentamicin [10].

**Figure 1:**
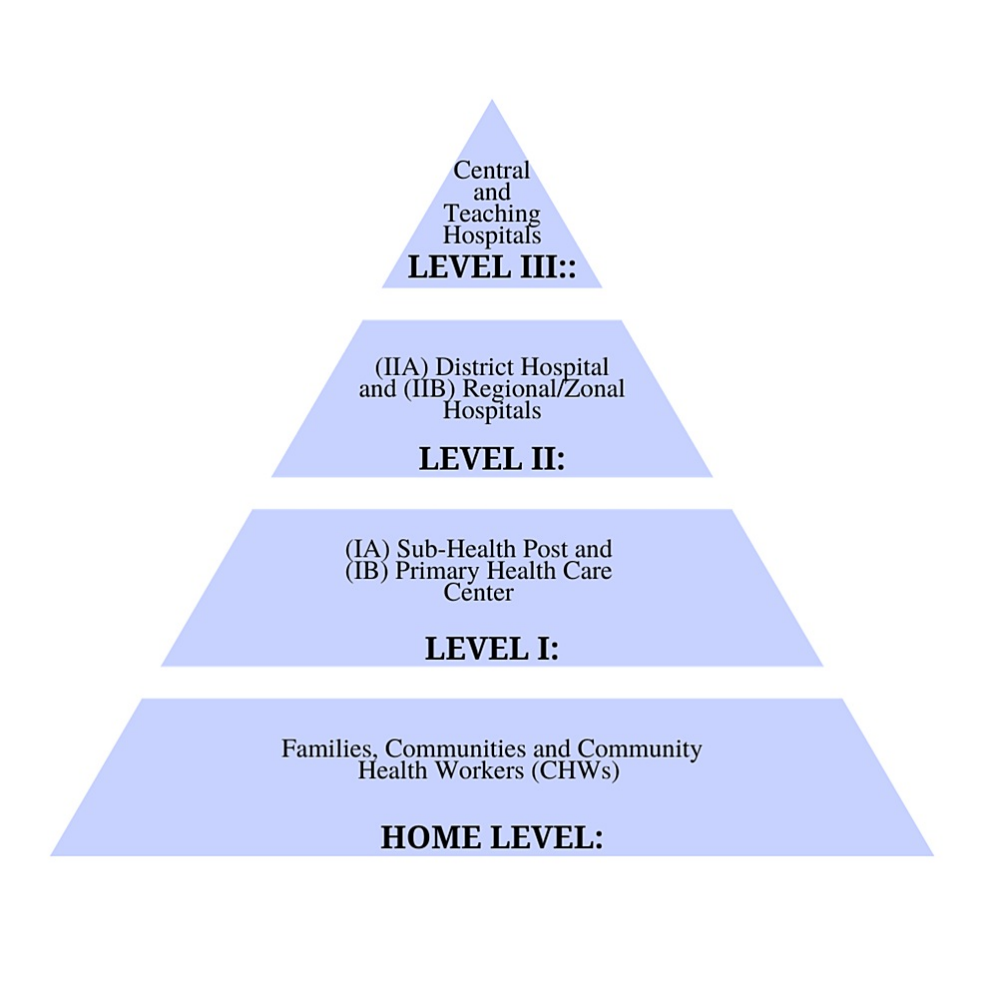
Levels of neonatal care establishment within the National Neonatal Health Strategy Adapted from [11]; open-access

Level IA neonatal care provides adequate referral and transport with thermal protection and supportive care for sick newborns. Level IB primary health center care is directed toward managing newborn infections, asphyxia, tube feeding, and monitoring Level 1A newborn care [10]. Level IIA, implemented in district hospitals, involves the management of complicated deliveries through IV therapy, obstetrical interventions, lumbar puncture, and basic laboratory services [10]. Level IIB, carried out in regional and zonal hospitals, provides specialist obstetric and neonatal care, incubator care, management of preterm neonates, and training for staff. Level III care, which is carried out in central and teaching hospitals, addresses surgical problems and the need for mechanical ventilation and provides parenteral nutrition and advanced imaging/laboratory services [10]. The pyramid that follows (Figure 1) illustrates these levels of care.

Extensive research by the MOHP informed that the major causes of neonatal death in Nepal may be attributable to infection, birth asphyxia, birth trauma, prematurity, hypothermia due to poor pregnancy health, inadequate pregnancy and delivery care, low birth weight, and poor postpartum care [11]. A vital component of the success of the NNHS has been the establishment of a referral system that provides appropriate care at varying levels of healthcare. However, without benchmark data to help provide efficacy of data, the effectiveness of this program is unclear. The NNHS provides essential opportunities to families by linking them to the community health level and further to the broader healthcare system, thus decreasing the time it takes to obtain adequate medical care.

Safe Delivery Incentives Program (SDIP) (Aama Surakshya Karyakram program)

Poor utilization of available healthcare resources is often due to high costs in Nepal [12]. Conditional Cash Transfer (CCT) programs, designed to promote healthcare utilization through cash incentives, have been initiated in several developing countries. The SDIP, also known as the Aama Surakshya Karyakram program, was implemented in 2005 in Nepal as a CCT program to decrease financial barriers and maternal mortality [12]. This program provides a monetary incentive to mothers for each infant delivery with the participation of a skilled birth attendant at home, in the hospital, or in a primary care health facility. The financial support varies according to geographical region; women in the Terai province are given 500 rupees, while in the Himalayan region, women receive 1500 rupees. An addendum to the program was made between 2009 and 2010 to provide an additional 400 Rupees for four completed antenatal visits, institutional delivery, and the first postnatal visit. The success of this program is evidenced by an increase of 3.2% in deliveries attended by a skilled healthcare worker per year. Additionally, in 2011, 35% of deliveries occurred in a hospital, and 36% of deliveries were in the attendance of skilled birth attendants (SBAs). This is a substantial increase when compared to prior years. Women who had participated in SDIP were 24% more likely to experience a government health facility delivery [13].

As expected, some challenges exist that have been revealed by surveying the population. The increase in hospital deliveries has led to long waiting times in the emergency rooms, due to bed shortages. Doctors have also reported increased levels of exhaustion and less time to manage lower-risk cases [14]. There have also been some concerns that mothers have not been receiving their incentives due to administrative delays, given that these funds were intended for travel and subsistence [15]. While more community mobilization is needed, the SDIP has provided an effective cash incentive process that promotes safe child delivery practices.

Free Newborn Care (FNC) services

The FNC service was initiated by the Government of Nepal in 2016 with the provision to treat sick newborns free of cost through all tiers of its healthcare delivery outlets. The goal of the program is to increase access to newborn care services to achieve the sustainable development goal of providing universal health coverage. This goal adds complementary newborn care services to lower barriers to health access associated with poverty [16].

This project was initiated after conducting five workshops in each SDG-designated region to assess newborn care services within public hospitals. By providing packages that meet the FNC guidelines, the cost of care is reimbursed for newborn care in birthing centers within the designated hospital [16]. As highlighted in Table 2, which illustrates the newborn care packages, compensation increases with the complexity of services provided.

**Table 2:** Free newborn care packages Adapted from [16]

Package	Services provided	Hospital reimbursement
Package 0 offered through birthing centers	Newborn resuscitation; kangaroo mother care; antibiotics provided based on the Integrated Management of Newborn and Childhood Illness criteria	No cost, provided through the government’s free health services
Package A offered through birthing centers	Antibiotics and other medications provided per the National Neonatal Clinical Criteria; basic laboratory services such as C-reactive protein, erythrocyte sedimentation rate, blood sugar, etc.; oxygen supplies; X-ray; ultrasound	1000 rupees
Package B offered through the Special Newborn Care Units (SNCUs)	Phototherapy; advanced laboratory services such as blood cultures and serum electrolyte levels; lumbar punctures; cerebrospinal fluid analysis; medications such as dopamine, dobutamine, phenytoin, etc.; bubble continuous positive airway pressure	2000 rupees
Package C offered through the Neonatal ICUs (NICUs)	Patients must be admitted to the NICU; bedside ultrasound and X-ray; labs such as arterial blood gases, serum electrolytes, osmolarity, and urine electrolytes; blood transfusions; volume exchange transfusions; mechanical ventilation	5000 rupees

FNC services aim to prevent the deprivation of healthcare to newborns due to poverty. One of the key barriers to overcome is out-of-pocket expenditure (OOPE), defined as direct payment for the cost of care. OOPE in many developing countries accounts for almost three-quarters or more of total expenditure on health. The FNC functions as a financing scheme to subsidize treatment for all sick newborns. It assures no-cost treatment of sick newborns admitted to the hospital by reimbursing charges for admission, bed, laboratory diagnosis, medications, and the doctor’s services [15]. 

A study conducted in 2018 reported that of 8,564 newborns who were admitted to 58 public hospitals, 1,573 received services from Package C, 3,722 received services from Package B, and 3,081 received services from Package A [16]. The main challenge faced by FNC hospitals has been coordinating care at the local level since many local hospitals have not started FNC services. Furthermore, the sustainability of this program remains a concern due to the cost of reimbursement and lack of human resources [16].

Unfortunately, a quasi-experimental study assessing OOPE and pre- and post-implementation of the FNC program in 12 public hospitals found no significant change in the treatment of sick newborns [15]. Other significant contributors to OOPE are the hospital stay of caregivers and the cost of travel from home [16]. These indirect costs are found to be higher than the direct cost of medical care [16]. By addressing these indirect costs, the FNC may be better able to achieve its aims. The main challenges faced in the implementation of healthcare services are lack of infrastructure, inadequate human resources, and poor reimbursement [17]. Enhancement of the FNC program has been proposed with due consideration to appropriate reimbursement and extra staffing [17]. This would provide opportunities to further promote the capacity building of healthcare workers and ensure the availability of newborn care.

Every Newborn Action Plan (ENAP)

The ENAP envisions no preventable deaths of newborns or stillbirths in Nepal, where every pregnancy is wanted, every birth celebrated, and women, babies, and children survive, thrive, and reach their full potential [18]. It was initiated by the Government of Nepal in 2016 with two goals: to reduce preventable newborn deaths in every province to less than 11 newborn deaths per 1,000 live births and to reduce preventable stillbirths in every province to less than 13 stillbirths per 1,000 total births by 2035 [19]. It has four strategic directions defined by the NHSS (2015-2020): equitable utilization of health services, quality for all, multisectoral approach, and reform [19]. Table 3 below outlines the goals and objectives of ENAP.

**Table 3:** Goals and objectives of ENAP BEMoNC: Basic Emergency Obstetric and Newborn Care; CEMoNC: Comprehensive Emergency Obstetric and Newborn Care; ENAP: Every Newborn Action Plan Adapted from [19]

Objective	Goals
Rebuild and restrengthen health systems	Develop new infrastructure under the guidance of the 2013 National Health Policy (one village, one health facility, 30 minutes within people’s reach); establish BEmONC services for every 10.000 people in the Terai region, birthing centers within two hours of people’s reach, and CEmONC services within six hours of reach; capacity building of such centers by reviewing and accelerating pre-service and in-service training, using e-learning, onsite visits, and mentoring of existing healthcare workers
Improve quality of care at the point of delivery	Develop national guidelines and training on the proper use of corticosteroids and tocolytics, conducting newborn healthcare training packages at different levels; expand the use of chlorhexidine for umbilical cord care; ensure timely referrals; implement quality improvement strategies (continued training, audits, and expanding current licensing authority); conduct site infection prevention training; ensure availability of functional waste management facilities
Incorporate newborn services into the Free Health Care Policy of Nepal	Improve access to health services and functionalize health service networks; implement national referral programs; introduce low-cost evidence-based interventions (the establishment of birthing centers, establishment of newborn care centers, expansion of lab services, provision of free transportation, and development of telemedicine)
Strengthen decentralized planning and budget	Work with local bodies for participatory planning, budgeting, and outlining respective targets for maternal and newborn health; build health facility operation committees
Improve sector management and governance	Ensure maternal and newborn health issue awareness programs targeted to underserved populations
Improve sustainability of health sector financing	Decrease the cost of newborn interventions; expand budget allocations for newborn and maternal health services; increase the efficiency of funds; provide information about the benefit packages to mothers
Improve healthy lifestyles and environment	Build equitable community engagement in planning; implement and monitor breastfeeding initiatives, adolescent and maternal nutrition, and social behavior communication strategies
Strengthen the management of public health emergencies	Better coordinate responses during and after disasters; improve rapid response teams; develop sub-national level protocols for maternal and newborn care during emergency situations
Improve the availability and use of evidence in the decision-making process	Advocate for effective use of civil registration and vital statistics; inform research at scaling evidence-based interventions; implement developing surveillance systems from the community level; measure impact and outcome measurements: maternal mortality rate, newborn mortality rate, stillbirth rate, under five years mortality rate, preterm birth rate, small for gestational age

One of the limitations of the ENAP intervention includes the lack of readily available impact and outcome measurements. Although mentioned as one of the goals of the program, there is no clear efficacy data. The ENAP is another strong initiative introduced by the Ministry of Health and remains one to be further evaluated.

Birth Preparedness Package (BPP)

The BPP, a part of the Safe Motherhood Plan initiatives through the MOHP, was developed to promote vital health information and induce behavior change. Implemented in 2003, this package encourages families, communities, and pregnant women to plan for deliveries, normal pregnancies, and postnatal care by implementing strategies for specific delay barriers, as outlined in Table 4 [17].

**Table 4:** BPP strategies to reduce delay barriers BPP: Birth Preparedness Package Adapted from [17]

Delay barriers in obstetric emergencies	BPP strategies to reduce delay barriers
Delay in problem recognition	Communication guidelines for CHWs for interactions with clients; flip-charts for CHWs and keychains for pregnant women that focus on four different areas of birth planning: antenatal care, intra and postpartum care for the mother and newborn, danger signs for women and newborn, financial preparations for birth and the postnatal period
Delay in searching for care	
Delay in receiving healthcare	

BPP utilizes three types of CHWs: (1) mobilizers consisting of FCHVs and SBAs, (2) supporters consisting of maternal child health workers (MCHWs), and (3) village health workers (VHWs). Both mobilizers and supporters undergo a two-day training on using BPP tools and effective counseling methods. The supporters are trained for an additional three days on techniques regarding safe motherhood. Supporters are expected to perform monthly assessments of the work outcomes of the mobilizers. Additionally, the FCHVs organize monthly discussions between mother’s groups to educate the community, using flip charts to communicate essential BPP messages. Individual counseling sessions are also provided [17]. Table 5 outlines the components of BPP.

**Table 5:** Components of the Birth-Preparedness Index (BPI) Adapted from [17]

Component number	Component
1	Received antenatal care at least one time from a qualified provider
2	Prolonged labor identified as a danger sign during delivery
3	Excessive bleeding identified as a danger sign during delivery
4	Financial preparations made for delivery emergencies
5	Preparations for emergency transportation during delivery
6	Delivery with a Skilled Birth Attendant (SBA) present
7	Postpartum care received by trained personnel within six weeks of delivery

Efficacy studies on the BPP in the Siraha district, Madhesh province, Nepal, indicated that essential newborn practices, which included activities such as breastfeeding and bathing, increased by 20-30%. According to the survey by McPherson et al., 30-48% of respondents were exposed to the program through keychains with messages on the essential areas of birth planning, and 58-64% of respondents interacted with a healthcare worker. Additionally, the birth-preparedness index (BPI) increased from 33% to 54% after program implementation [17]. A statistically significant increase was noted in six of seven components of the BPI. However, the use of a skilled birth attendant during delivery remained unchanged from baseline. Furthermore, 59% of the respondents did not think it was necessary for them to attend monthly discussions. While an increase in education and implementation of these strategies was noted, the BPP remains a program that requires additional promotion and improvement to be widely used throughout Nepal [17].

Future implications

Based on current data and programs available, it is evident that Nepal has made significant progress in its goals of reducing child and infant mortality. From 1990 to 2020, Nepal has reduced the under-five mortality rate from 138.8 deaths per 1,000 live births to 28.2 deaths per 1,000 live births. To achieve further goals laid out by the Nepal MOHP, the main challenge for Nepal is to decrease neonatal mortality rates. Nepal’s neonatal mortality rate is currently 17 deaths per 1,000 live births, a marked decrease from 57.92 deaths per 1,000 live births in 1990 [20]. However, the rate of reduction has remained relatively stagnant over the past few years. Nepal is still on track to achieve the SDGs laid out by the WHO. The positive trend in the reduction of neonatal mortality in Nepal may be linked to its programs aimed at addressing antenatal and postnatal care, family planning, and improving safe delivery care practices. Additionally, the use of FCHVs plays a crucial role in strengthening care in Nepal’s public health system. A greater capacity of providers at all healthcare levels, especially in rural areas, should greatly improve maternal and newborn care [2].

Over the past 20 years, Nepal has implemented several programs to address maternal and child health across the nation. However, a recurring limitation presented by these programs is the availability of up-to-date efficacy data. Due to the broad range of programs implemented and ongoing interventions, it is difficult to attribute a reduction in mortality rates to any single program or component. Furthermore, data available in different regions of the country varies greatly and may be attributed to socio-economic disparities in rural areas. Due to the significant monetary and personnel resources allocated to these programs, it would be helpful for the MOHP to implement an effective evaluation of these programs [2]. Additionally, the majority of these programs such as the SDIP and NNHS focus on increasing the medical knowledge and skills of healthcare workers to prevent and diagnose newborn illnesses while promoting community awareness of health services. Additional efforts should be employed to strengthen the healthcare facilities themselves by providing basic medical supplies and medications for the care of newborns and tools to screen the newborns for potential illnesses.

## Conclusions

Limitations that should be considered when interpreting this study include the potential that pertinent literature on these screening programs may have been missed. Literature may have been excluded due to English-only language articles. Only searching for literature from 2004 onwards may have also excluded some studies. Excluding terms such as “prevention”, “neonatal death prevention”, “infant death prevention”, “neonatal mortality prevention”, and “infant mortality prevention” may have resulted in relevant literature being excluded. Additional literature may have been produced by expanding this search criteria. Lastly, due to the nature of the topics, some of the research obtained was from governmental documentation, which did not appear to be peer-reviewed.

Overall, infant screening is an essential public health measure. Nepal has made significant strides in combating infant and child mortality as evidenced by the decrease in under-five mortality rate per 1,000 live births and neonatal mortality rate.
